# Prognostic Value of Tumor Markers, NSE, CA125 and SCC, in Operable NSCLC Patients

**DOI:** 10.3390/ijms140611145

**Published:** 2013-05-27

**Authors:** Dangfan Yu, Kaiqi Du, Taifeng Liu, Guojun Chen

**Affiliations:** 1 Department of Nuclear Medicine, Zhejiang Provincial Corps Hospital, Chinese People’s Armed Police Force, Jiaxing 314000, China; E-Mail: vip20122013@126.com; 2 Department of Thoracic Surgery, Zhejiang Provincial Corps Hospital, Chinese People’s Armed Police Force, Jiaxing 314000, China; E-Mail: dukaiqi1111@126.com; 3 Department of Laboratory Medicine, Zhejiang Provincial Corps Hospital, Chinese People’s Armed Police Force, Jiaxing 314000, China; E-Mail: zlzwanger@126.com

**Keywords:** tumor marker, CA125, NSE, SCC, lung cancer

## Abstract

The aim of this study was to investigate the prognostic value of tumor markers in operable non-small cell lung cancer (NSCLC) patients. A total of 481 NSCLC patients were enrolled in the present study. High levels of neuron-specific enolase (NSE), carbohydrate antigen 125 (CA125) and squamous cell carcinoma antigen (SCC) were detected in 306 (63.6%), 89 (18.5%) and 125 (26.0%) patients, respectively. Seventy-eight of 481 patients died of disease progression, and the median disease-free survival (DFS) and overall survival (OS) were 16.0 and 21.0 months, respectively. The three-year DFS rate was 56.7%, and the OS rate was 75.3%. For serum NSE, the three-year cumulative DFS rate for the normal and elevated group was 67.7% and 51.8% (*p* = 0.007). The OS in patients with high and normal levels of NSE was 34.0 months and 48.0 months, respectively. The median DFS was 46.0 months *versus* 32.0 months (*p* = 0.001), and the OS was 48.0 months *versus* 44.0 months (*p* = 0.001) in patients with normal and high levels of CA125. For patients with squamous cell carcinoma, the overall survival was significantly shorter in patients with elevated levels of SCC (*p* = 0.041). In the multivariate analysis high levels of NSE, CA125 and clinical stage were significantly correlated with worse prognosis (*p* < 0.05). Patients with all three tumor markers elevated presented the worst prognosis (*p* < 0.05). In our analysis, high levels of preoperative serum NSE and CA125 are correlated with worse survival in operable NSCLC patients.

## 1. Introduction

Lung cancer is the most common malignant neoplasm in the world. Non-small cell lung cancer (NSCLC) represents 80%–85% of patients diagnosed with lung cancer [1]. Radical surgery has been the standard treatment for many decades. Tumor stage at diagnosis is the most important prognostic factor to predict survival. Unfortunately, the majority of patients are newly diagnosed with NSCLC at a late stage [2], resulting in a high mortality rate. A variety of strategies that adopt a combination of chemotherapy and radiotherapy have been investigated in patients with advanced disease post operation. However, a great deal of patients still experienced disease progression in a short time, exhibiting a five-year survival rate of approximately 15% [3].

Generally, tumor size detected by imaging or physical examination is considered to be the gold method for evaluating the efficacy of treatment. However, treatment response can’t be assessed in operable NSCLC patients, because they don’t have measurable masses post operation. Serum tumor biological markers could be taken into account as supplement [4]. However, none of these had been proven to be sufficiently and effectively for clinical use. Most of these markers were somewhat controversial and inconclusive [5]. Neuron-specific enolase (NSE), carbohydrate antigen 125 (CA125) and squamous cell carcinoma antigen (SCC) are three markers commonly regarded as tumor markers in NSCLC. NSE has been widely regarded as a marker of small cell lung cancer [6]. A high level of NSE could be observed in some NSCLC and might be correlated with poor prognosis [7,8]. CA125, a mucinous glycoprotein, has been used in the diagnosis and follow-up of ovarian cancer [9,10]. A significant correlation between CA125 and the outcome of NSCLC has been reported [11]. SCC originally obtained from squamous cell carcinoma tissue from the uterine cervix [12] has been proven to be a well-established tumor marker for squamous cell carcinoma [13]. However, many reports discouraged its clinical routine use in NSCLC, due to its low sensitivity [14,15].

In the present study, we designed a retrospective clinical analysis of a total of 481 operable NSCLC patients to investigate the relationship between these three tumor markers and patients’ characteristics and prognosis.

## 2. Results and Discussion

### 2.1. Patients’ Characteristics

A total of 481 NSCLC patients were enrolled in the present study. The patients’ characteristics are summarized in Table 1. According to the criteria of the World Health organization/International Association for the Study of Lung Cancer (WHO/IASLC) classification of lung tumors, 236 were squamous cell carcinoma, 221 were adenocarcinoma and 24 were large cell carcinoma; 27 were well differentiated, 248 were moderately differentiated and 206 were poorly differentiated. There were 336 cases with smoking. In terms of the new IASLC staging system, 217 cases were categorized as stage I, 120 as stage II and 144 as stage III.

### 2.2. Tumor Markers and Patients’ Characteristics

The relationship between patients’ characteristics and tumor markers is shown in Table 1. The median serum level of NSE was 18.4 ng/mL (3.4–344.2 ng/mL) for the entire population. Three-hundred and six (60.0%) patients had NSE more than or equal to 12.5 ng/mL, defined as high range. There was no significant correlation among NSE and sex, age, smoking status, tumor histologic type and cancer cell differentiation. We detected significant correlation between NSE and T stage (*p* = 0.000). Of the 481 patients analyzed for CA125, 89 patients (17.5%) had elevated levels (CA125 ≥ 35 U/mL). The median level of CA125 was 14.4 U/mL (range: 2.5–460.1 U/mL). There was a significant relationship between cancer cell differentiation and CA125 levels (*p* = 0.021). The T stage, N stage and the clinical stage were also correlated with CA125 (*p* < 0.05). The median level of SCC was 1.00 ng/mL (0.3–41.7 ng/mL). There was a statistically significant correlation between SCC and tumor histologic type (*p* = 0.000). A high level of SCC was detected in male patients (*p* = 0.000). However, no difference in SCC levels was detected according to the N stage and clinical stage (*p* > 0.05).

### 2.3. Association of Tumor Markers with Disease-Free Survival and Overall Survival

**Figure 1:**
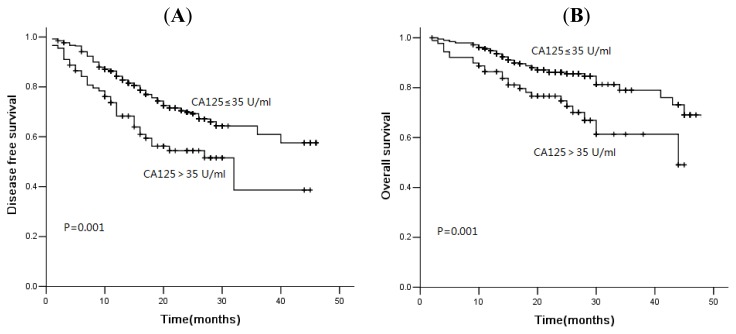
Kaplan-Meier disease-free survival curves (**A**) and overall survival curves (**B**) according to CA125: patients with high levels of CA125 showed shorter disease-free survival and overall survival.

**Figure 2:**
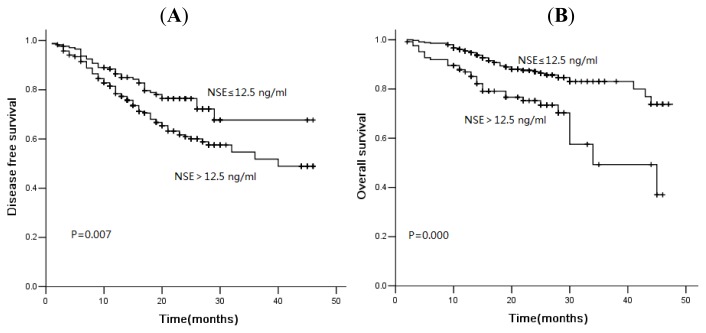
Kaplan-Meier disease-free survival curves (**A**) and overall survival curves (**B**) according to NSE: patients with high levels of NSE showed shorter disease-free survival and overall survival.

**Figure 3:**
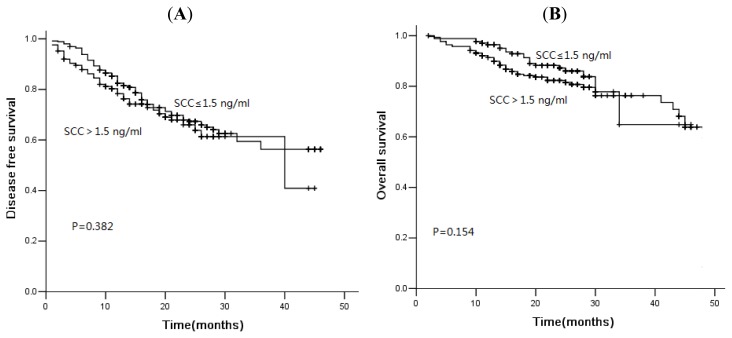
Kaplan-Meier disease-free survival curves (**A**) and overall survival curves (**B**) according to SCC: The serum level of SCC was not associated with disease-free survival (DFS) or overall survival (OS).

**Figure 4:**
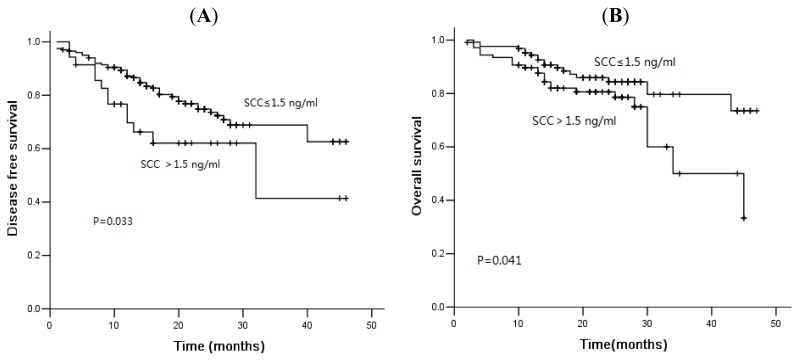
Kaplan-Meier disease-free survival curves (**A**) and overall survival curves (**B**) according to SCC in squamous cell lung cancer: patients with high levels of SCC showed shorter overall survival.

In the current study, 78 of 481 patients died of disease progression, and the median DFS and OS were 16.0 and 21.0 months, respectively. The three-year DFS rate was 56.7%, and the OS was rate 75.3%. The median PFS was 46.0 months *versus* 32.0 months (*p* = 0.001), and the OS was 48.0 months *versus* 44.0 months (*p* = 0.001) in patients with normal and high levels of CA125 (Figure 1). Similarly, for serum NSE, the three-year cumulative DFS rate for normal and elevated group was 67.7% and 51.8% (Figure 2, *p* = 0.007). The median OS of patients with normal levels and elevated levels was 48.0 months and 34.0 months, respectively. There was a significant difference between these two groups (Figure 2, *p* = 0.000). The serum levels of SCC were not associated with DFS or OS (Figure 3, *p* > 0.05). However, for patients with squamous cell carcinoma, the overall survival was significantly shorter in patients with elevated levels of SCC (Figure 4, *p* = 0.041).

In a multivariable Cox regression model, advanced clinical stage, serum CA125 ≥ 35 U/mL and serum NSE ≥ 12.5 ng/mL were the independent factors associated with significantly unfavorable disease-free survival (Table 2). Furthermore, age ≥65 year, advanced clinical stage, serum CA125 ≥ 35 U/mL and serum NSE ≥ 12.5 ng/mL were the independent factors associated with significantly unfavorable overall survival (Table 2). In addition, Cox proportional hazards regression showed that serum SCC was an independent prognostic factor in operable NSCLC. Patients who had serum SCC ≥ 1.5 ng/mL had an elevated risk of disease progression and death compared with patients who had serum SCC < 1.5 ng/mL. The hazard ratio (HR) was 4.067 (95% confidence interval [CI], 1.639–10.091) for disease progression and 6.909 (95% CI, 2.167–22.026) for death, and the trend linking increasing fibrinogen levels with risk also was statistically significant for both outcomes (*p* < 0.05) (Table 3).

**Figure 5:**
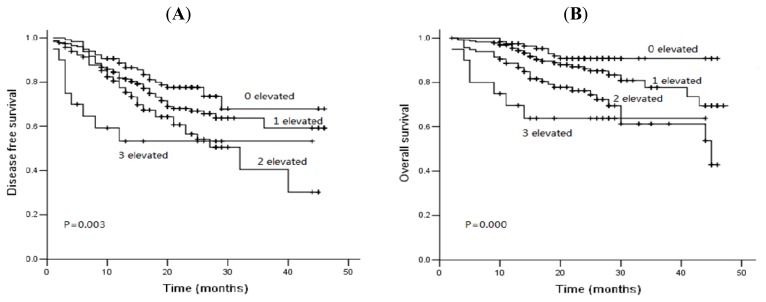
Kaplan-Meier disease-free survival curves (**A**) and overall survival curves (**B**) according to the score number of three tumor markers combined.

We analyzed the prognostic value of combination of these three tumor markers. We observed that in this study, 20 patients (4.2%) presented three elevated markers, 116 patients (24.1%), two elevated markers, and 117 patients (24.3%) three normal markers. We compared DFS and OS between these four groups. Patients with three elevated markers proved to have a significantly shorter DFS and OS (Figure 5, *p* < 0.05).

### 2.4. Discussion

Tumor markers are frequently used in clinical practice. However, no serum tumor marker is both sensitive and specific for NSCLC. The results in the present study indicated that pre-operative serum NSE levels could be used as a biomarker for outcome prediction in non-small cell lung cancer. Elevated serum NSE levels were correlated with worse prognosis in NSCLC patients. To the best of our knowledge, this is the largest sample report to reveal a correlation between serum NSE levels and the prognosis of operable NSCLC patients.

Although the prognostic value of NSE in SCLC had been widely accepted [16,17], the value in NSCLC was controversial. In the Pujol *et al.* study with 621 all stages NSCLC patients (majority local advanced or metastatic NSCLC), NSE was a prognostic factor for survival [18]. Similar results were found by other studies, but the sample of the study was relatively small [19,20]. In 231 patients with brain metastasis, elevated NSE levels were found to be a prognostic determinant for overall survival [21]. In our study, high serum NSE levels were correlated with worse prognosis in NSCLC patients, consistent to the results in these studies. However, in the study from Reinmuth *et al.* [22], with 67 operable early stage NSCLC patients, preoperative serum NSE levels were not associated with prognosis.

The prognostic role of CA125 in NSCLC was not elucidated. Díez M. *et al.* [23] once reported that preoperative serum CA125 levels were related with TNM stage in operable NSCLC and could provide additional prognostic information. Serum CA125 levels were found to be a tool to monitor the tumor recurrence and disseminated failure post operation [24]. Similar results were found by other authors [25,26]. However, in some case, elevated serum CA125 did not relate with tumor recurrence [27]. In our present study, elevated serum CA125 levels were associated with tumor cell differentiation, TNM stage and survival. Furthermore, in a multivariable Cox regression model, CA125 was found to be an independent factor associated with survival.

Many clinicians investigated the value of SCC in the clinical management of NSCLC patients. In the study of Foa *et al.* [28] with 62 resectable NSCLC, serum SCC levels have good prognostic ability. Mizuguchi and colleagues [29] have examined the prognostic role of serum SCC levels in stage I NSCLC patients and draw a conclusion that SCC levels were significantly associated with survival. However, in the multivariate analysis, serum SCC levels were not independent prognostic factors. Furthermore, not all studies were consistent with the results in these studies. In two small sample studies [22,30], a lack of association between serum SCC levels and prognosis was observed. In our study with 510 operable NSCLC patients, serum SCC levels were not correlated with survival. However, in squamous cell lung cancer patients, serum SCC levels paly a strong prognostic role.

Despite extensive studies describing the role of tumor markers in the diagnosis of NSCLC, most results still remain debated. None of these biomarkers are mature enough to be routinely used in clinical practice. Combining many tumor biomarkers appropriately increases sensitivity and helps with the diagnosis of NSCLC [31]. Chiu *et al.* reported that the change in tumor markers (combination of carcinoembryonic antigen (CEA), CA125 and CA199), before and after gefitinib-based chemotherapy, was closely related to the tumor response and progression-free survival [32]. However, in early stage NSCLC, the value of NSE, CA125 and SCC was limited, even a combination of these three markers [33]. Serum CEA might be another promising tumor marker for NSCLC [34]. The use of tumor marker scores, including CEA, might possibly be an important approach in diagnosis and predicting the outcome of NSCLC [35].

There are some limitations in our present study. In this study, we did not consecutively investigate the serum tumor markers’ levels post operation and during the follow-up or for the recurrence assessment. The relationship between changes in tumor markers and tumor progression need to be investigated. Second, our study included a comparative homogeneous population with the majority of male and smoker patients, which might cause a bias. Also, this is a retrospective study based on patients of one center and could not completely avoid selection bias.

The recent advances in molecular biology have led to understanding the genetics of lung cancer and predicting the outcome of lung cancer patients. The roles of these biologic markers during diagnosis, treatment and follow-up have been the subject of extensive studies recently [36]. Evaluation of these tumor markers might be an earliest clue to tumor progression and may provide the possibility of treatment. The results of our study showed that high levels of preoperative serum NSE and CA125 are correlated with worse survival in operable NSCLC patients. NSE, CA125 and SCC, when measured pre-operation, may provide additional information for prognosis of patients with NSCLC.

## 3. Experimental Section

### 3.1. Patients and Treatment

The present study enrolled 481 patients who had been diagnosed as having non-small cell lung cancer, between 2006 and 2009, at Zhejiang Provincial Corps Hospital, China. All patients had bad been newly confirmed as NSCLC, without previous treatment. Patients with previous or coexisting cancer other than NSCLC were excluded from this study. The study population had a median age of 60 years (range: 37 to 82 years). The patients comprised of 364 (75.7%) males and 117 (24.3%) females. Approval for the present study was obtained from the institutional review board of the hospital. All patients provided informed consent prior to undergoing surgery.

All patients experienced surgery. Lobectomy, bilobectomy or pneumonectomy was performed according to the location or size of lung neoplasm. Systematic mediastinum lymph node dissection was performed in all patients. 223 patients were treated with adjuvant platinum-based chemotherapy or adjuvant radiotherapy or a combination of the two. Detailed information about patient’s characteristics and tumor histopathology was collected retrospectively from the medical records.

### 3.2. Tumor Markers Measurement

A blood sample was obtained by peripheral venous puncture, taken before breakfast, 3 days prior to the surgery. Serum levels of NSE, CA125 and SCC were measured by enzyme-linked immunosorbent assay (ELISA). According to the manufacturer’s instructions and previously published results [11,13,18], the upper limits of the normal values were at 12.5 ng/mL for NSE, 35 U/mL for CA125 and 1.5 ng/mL for SCC.

### 3.3. Follow-Up

All patients received standardized follow-up, occurring at a 3 month interval for two years, a 6 month interval the third year and yearly thereafter. Evaluation comprised a physical examination, complete blood count, chest computed tomography (CT), brain magnetic resonance imaging (MRI) and abdominal ultrasound. Local recurrence and distance metastasis was histologically confirmed whenever possible.

### 3.4. Statistical Analysis

The chi-square test was performed to evaluate the association between clinicopathological variables and tumor markers. Disease-free survival (DFS) was defined from the date of definitive surgery to the date of local or distant progression, death of any cause or the date of last follow-up. Overall survival (OS) was calculated as the time from pulmonary surgery to death or censoring. Kaplan-Meier curves were used to estimate the distribution of DFS and OS, and a two-sided log-rank test was performed to compare the difference between survival curves. We used the Cox proportional hazards model with the backward selection method for multivariate analysis. All factors with effects on DFS and OS in univariate analysis (*p* ≤ 0.10) were included in the multivariate analysis. All statistical calculations were performed with SPSS 13.0 for Windows (Chicago, IL, USA). A *p*-value of less than 0.05 was considered statistically significant.

## 4. Conclusions

In summary, preoperative serum NSE and CA125 levels could help us predict the prognosis of NSCLC patients. Further, a large and prospective study is needed to warrant this.

## Figures and Tables

**Table 1:** Association of tumor marker with parameters of patients with non-small cell lung cancer (NSCLC).

Variables	Patient *n* (%)	NSE level		*p*	CA125 level		*p*	SCC level		*p*
		
		Normal	High		Normal	High		Normal	High	0.000
Sex				0.752			0.652	246 (67.6)	118 (62.4)	
Male	364 (75.7)	131 (36.0)	233 (64.0)		295 (81.0)	69 (19.0)		110 (94.0)	7 (6.0)	
Female	117 (24.3)	44 (37.6)	73 (62.4)		97 (82.9)	20 (17.1)				

Age				0.206			0.092			0.917
<65	333 (69.2)	115 (34.5)	218 (65.5)		278 (83.5)	55 (16.5)		246 (73.9)	87 (26.1)	
≥65	148 (30.8)	60 (40.5)	88 (59.5)		114 (77.0)	34 (23.0)		110 (74.3)	38 (25.7)	

Smoking				0.643			0.640			**0.000**
Never	145 (30.1)	55 (37.9)	90 (62.1)		120 (82.8)	25 (17.2)		130 (89.7)	15 (10.3)	
Ever/Current	336 (69.9)	120 (35.7)	216 (64.3)		272 (81.0)	64 (19.0)		226 (67.3)	110 (32.7)	

Alcohol				0.403			0.104			**0.001**
Never	249 (48.8)	95 (38.2)	154 (61.8)		196 (78.7)	53 (21.3)		200 (80.3)	49 (19.7)	
Ever/Current	232 (48.2)	80 (34.5)	152 (65.5)		196 (84.5)	36 (15.5)		156 (67.2)	76 (32.8)	

Histologic type				0.835			0.762			**0.000**
Squamous cell	236 (49.1)	84 (35.6)	152 (64.4)		195 (82.6)	41 (17.4)		128 (54.2)	108 (45.8)	
Adenocarcinoma	221 (45.9)	81 (36.7)	140 (63.3)		177 (80.1)	44 (19.9)		209 (94.6)	12 (5.4)	
Others	24 (5.0)	10 (41.7)	14 (58.3)		20 (83.3)	4 (16.7)		19 (79.2)	5 (20.8)	

Differentiation				0.495			**0.021**			**0.001**
Well	27 (5.6)	12 (44.4)	15 (55.6)		25 (92.6)	2 (7.4)		22 (81.5)	5 (18.5)	
Moderate	248 (51.6)	93 (37.5)	155 (62.5)		210 (84.7)	38 (15.3)		166 (66.9)	82 (33.1)	
Poor	206 (42.8)	70 (34.0)	136 (66.0)		157 (76.2)	49 (23.8)		168 (81.6)	38 (18.41)	

T stage				**0.000**			**0.001**			**0.039**
T1	47 (9.8)	24 (51.1)	23 (48.9)		43 (91.5)	4 (8.5)		41 (87.2)	6 (12.8)	
T2	342 (71.1)	130 (38.0)	212 (62.0)		285 (83.3)	57 (16.7)		255 (74.6)	87 (25.4)	
T3	57 (11.9)	6 (10.5)	51 (89.5)		43 (75.14)	14 (24.6)		36 (63.2)	21 (36.8)	
T4	35 (7.3)	15 (42.9)	20 (57.1)		21 (60.0)	14 (40.0)		24 (68.6)	11 (31.4)	

N stage				0.360			**0.004**			0.223
N0	251 (52.2)	97 (38.6)	154 (61.4)		218 (86.9)	33 (13.1)		194 (77.3)	57 (22.7)	
N1	128 (26.6)	40 (31.3)	88 (68.8)		100 (78.1)	28 (21.9)		91 (71.1)	37 (28.9)	
N2	102 (21.2)	38 (37.3)	64 (62.7)		74 (72.5)	28 (27.5)		71 (69.6)	31 (30.4)	

Clinical stage				0.092			**0.000**			0.189
I	217 (45.1)	90 (41.5)	127 (58.5)		191 (88.0)	26 (12.0)		171 (78.8)	46 (21.2)	
II	120 (24.9)	36 (30.0)	84 (70.0)		97 (80.8)	23 (19.2)		84 (70.0)	36 (30.0)	
IIIa	109 (22.7)	34 (31.2)	75 (68.8)		83 (76.1)	26 (23.9)		77 (70.6)	32 (29.4)	
IIIb	35 (7.3)	15 (42.9)	20 (57.1)		21 (60.0)	14 (40.0)		24 (68.6)	11 (31.4)	

Bold values are statistically significant (*p* < 0.05). NSE, neuron-specific enolase; CA125, carbohydrate antigen 125; SCC, squamous cell carcinoma antigen.

**Table 2:** Multivariate analysis for disease free survival (DFS) and overall survival (OS) for all patients.

End point	Parameter	HR	95% CI	*p*
DFS	Sex: Male *vs.* Female	1.005	0.528–1.914	0.988
	Age: <65 Y *vs*. ≥65 Y	1.379	0.971–1.959	0.072
DFS	Smoking: Ever *vs*. Never	1.030	0.573–1.850	0.922
DFS	Clinical stage: I, II *vs*. III	1.298	1.093–1.542	**0.003**
DFS	NSE level: <12.5 ng/mL *vs*. ≥12.5 ng/mL	1.609	1.110–2.333	**0.012**
DFS	CA125 level: <35 U/mL *vs*. ≥35 U/mL	1.857	1.121–2.407	**0.006**
DFS	SCC level: <1.5 ng/mL *vs*. ≥1.5 ng/m	1.236	0.805–1.896	0.333

OS	Sex: Male *vs*. Female	0.820	0.308–2.180	0.690
	Age: <65 Y *vs*. ≥65 Y	1.676	1.051–2.673	**0.030**
OS	Smoking: Ever *vs*. Never	1.111	0.474–2.604	0.808
OS	Clinical stage: I, II *vs*. III	1.377	1.089–1.743	**0.003**
OS	NSE level: <12.5 ng/mL *vs*. ≥12.5 ng/mL	1.907	1.148–3.169	**0.013**
OS	CA125 level: <35 U/mL *vs*. ≥35 U/mL	2.042	1.290–3.225	**0.005**
OS	SCC level: <1.5 ng/mL *vs*. ≥1.5 ng/mL	1.303	0.788–2.157	0.303

Bold values are statistically significant (*p* < 0.05).

**Table 3:** Multivariate analysis for disease-free survival (DFS) and overall survival (OS) in squamous cell lung cancer.

End point	Parameter	HR	95% CI	*p*
DFS	Sex: Male *vs*. Female	1.527	0.687–3.393	0.299
	Age: <65 Y *vs*. ≥65 Y	1.135	0.659–1.955	0.648
DFS	Smoking: Ever *vs*. Never	1.764	0.786–3.959	0.169
DFS	Clinical stage: I, II *vs*. III	2.154	1.256–3.695	**0.005**
DFS	NSE level: <12.5 ng/mL *vs*. ≥12.5 ng/mL	1.205	0.725–2.004	0.471
DFS	CA125 level: <35 U/mL *vs*. ≥35 U/mL	1.459	0.841–2.531	0.179
DFS	SCC level: <1.5 ng/mL *vs*. ≥1.5 ng/m	4.067	1.639–10.091	**0.002**

OS	Sex: Male *vs*. Female	1.609	0.395–6.552	0.507
	Age: <65 Y *vs*. ≥65 Y	2.242	0.908–5.533	0.080
OS	Smoking: Ever *vs*. Never	1.857	0.443–7.779	0.397
OS	Clinical stage: I, II *vs*. III	2.515	1.105–5.723	**0.028**
OS	NSE level: <12.5 ng/mL *vs*. ≥12.5 ng/mL	2.007	0.804–5.012	0.136
OS	CA125 level: <35 U/mL *vs*. ≥35 U/mL	0.715	0.300–1.706	0.450
OS	SCC level: <1.5 ng/mL *vs*. ≥1.5 ng/mL	6.909	2.167–22.026	**0.001**

Bold values are statistically significant (*p* < 0.05).
